# The American Leech Trade

**Published:** 1877-02

**Authors:** 


### Selections.

### THE AMERICAN LEECH TRADE.

One of the oldest American leech dealers has been interviewed by the correspondent of a cotemporary. His opinions are as follows:—

"The American leech I believe to be utterly valueless. I have received fine looking specimens from Mississippi and Pennsylvania, but I found them wholly worthless. They are far inferior to even some European varities of the hirudo decora, which can not easily be induced to bite unless blood be drawn to excite them. I consider six Swedish leeches equal to at least one hundred of any American variety. Those exported to America are generally full of blood, and at Rhode Island there are immense purging ponds in which the newly arrived leeches are placed, and left to digest their last meal. Until it has been perfectly digested they are useless. These ponds belong to Mr. Witte, who does nearly all the importing for American leech doctors, and he charges an extra price for the ponded leech, because the leeches must remain at least a year in the purging pond. It takes a year for them to get rid of one good meal. The leech can live on almost nothing; its vitality is absolutely prodigious; it has been known to live in the human stomach, and to make its home in the human intestines. They live to a prodigious age, from fifty to one hundred years. But it is a curious thing that they are constitutionally delicate creatures. If deprived for a considerable time of clay or turf to burrow in, they are liable to disease. They are carried off by epidemics peculiar to leech life, some of which appear to be skin diseases. I have to nurse them pretty carefully, and when I find one leech sick I put him in the leech hospital. A milk diet frequently restores sick leeches to perfect health."

